# Combined minimally invasive vagal cranial nerve and trigeminocervical complex peripheral nerve stimulation produces prolonged improvement of severe painful peripheral neuropathy and hyperglycemia in type 2 diabetes

**DOI:** 10.3389/fnins.2025.1644961

**Published:** 2025-08-26

**Authors:** Peter S. Staats, Alyssa Staats, Brittny Mikhaiel, Jason Chen, Eric Azabou, Claire-Marie Rangon

**Affiliations:** ^1^Vagus Nerve Society, Florida, FL, United States; ^2^National Spine and Pain Centers, Florida, FL, United States; ^3^Fraym, Arlington, VA, United States; ^4^United States Air Force, California, CA, United States; ^5^George Washington University School of Medicine, Washington, DC, United States; ^6^Clinical Neurophysiology and Neuromodulation Unit, Department of Physiology, Raymond Poincaré Hospital, Assistance Publique-Hôpitaux de Paris, Paris, France; ^7^Laboratory of Infection and Inflammation Inserm, University of Versailles Saint-Quentin en Yvelines, Paris-Saclay University, Paris, France

**Keywords:** vagus nerve, trigeminocervical complex, minimally invasive stimulation, closed-loop neuromodulation, type 2 diabetes, diabetic peripheral neuropathy, glucagon-like peptide-1

## Abstract

**Introduction:**

Diabetic Peripheral Neuropathy (DPN), a debilitating complication of type 2 diabetes mellitus (T2DM), stems from bioenergetic failure and reduced vascular endothelial growth factor-A expression (VEGF-A), persisting despite optimal glycemic control. The meteoric rise of “diabesity”—the coexistence of obesity and T2DM—underscores the ongoing failure of symptom control strategies and the critical need to immediately address the root cause of metabolic dysfunction and neuropathic pain.

**Methods:**

An analysis was performed on patients who received combined minimally invasive auricular vagus cranial nerve stimulation (aVNS) and trigeminocervical complex (TCC) peripheral nerve stimulation in 83 Native American patients (91 initial, 8 lost to follow-up) with severe T2DM and DPN pain who were offered stimulation in the routine course of clinical care. Participants were implanted on branches of their vagal and trigeminal cranial nerves, along with their upper cervical peripheral nerves and stimulated for 19 days prior to explantation. Numerical Rating Pain Scores (NRS) and mean blood glucose levels were measured at 30-, 60-, and 90-days post-explant.

**Results and discussion:**

Notable results include: NRS pain scores dropping 87% (7.92 to 1.04), mean blood glucose decreasing 37% (209 to 121 mg/dL), and HbA1c levels falling from 8.9% to 5.8% at 90 days. These improvements were all sustained for an average of 7.85 months of follow up. Additionally, a random subset decreased 80% of all pain and diabetes medications. This efficacy surpasses prior outcomes from cervical VNS alone, highlighting the synergy of targeting both the vagal and trigeminal cranial nerves along with the trigeminocervical complex.

**Discussion:**

These findings position combined minimally invasive aVNS and TCC peripheral nerve stimulation as a promising immediate therapy for the current DPN and diabesity crisis, as well as a potential non-pharmacologic alternative for the management of type 2 diabetes.

## Introduction

Diabetes is a major healthcare crisis affecting more than 422 million people worldwide with 38 million in the USA—15% of which have painful Diabetic Peripheral Neuropathy (DPN). The direct cost of diabetes (including pain) has been steadily increasing—412B in 2022 in the United States (US) alone (ADA 2022 report)—not including the 95 million pre-diabetics and their combined associated co-morbidities costs. At the current rate, the overall cumulative cost of diabetes, pre-diabetes and its associated co-morbidities will consume all US healthcare expenditures within the next 5–10 years (Dyess, 2023; Lin et al., 2018; Cawley et al., 2021). This meteoric rise underscores the failure of current symptom control strategies and the critical need for immediate therapies addressing the root cause of the underlying metabolic dysfunction and associated co-morbidities including neuropathic pain.

Type two diabetes (T2DM) appears to have a genetic predisposition and typically affects those with a poor diet and obesity. Native Americans are 2.3 times more likely to die from complications of diabetes compared to the general American population. DPN is a debilitating neurologic disorder characterized by a distal-to-proximal loss of peripheral nerve function, associated with pain, disability, as well as higher all-cause and cardiovascular mortality (Hicks et al., 2021). Sensory neurons, vulnerable to hyperglycemic damage due to their lack of insulin-regulated glucose uptake can still be affected in well-controlled T2DM (Tomlinson and Gardiner, 2008). Reduced expression of vascular endothelial growth factor-A (VEGF-A) underlies neuronal damage and pain in DPN, as VEGF-A supports both vascular and neuronal integrity (Jerić et al., 2017; Pawson et al., 2010; Carmeliet and Storkebaum, 2002). Since the VEGF family also regulates lipid metabolism (Zhou et al., 2023), additional metabolic risk factors, including dyslipidemia and metabolic syndrome, exacerbate peripheral nerve bioenergetic failure. Notably, the term “diabesity,” coined over 50 years ago, underscores the pathophysiological link between obesity and T2DM as well (Sims et al., 1973).

DPN management in T2DM patients has traditionally centered on lifestyle interventions including weight loss, exercise, systemic and topical medications and implanted single modality neuromodulation therapies. Current therapeutic approaches often fall short in providing sustained symptom relief, necessitating the exploration of alternative modalities that address the root cause of metabolic and neurovascular dysfunction (Barrett et al., 2007; Yang et al., 2022). More recently neuromodulation strategies including spinal cord stimulation and auricular stimulation have been approved by the FDA for diabetic neuropathy pain.

Even with aggressive worldwide symptom control pharmacological strategies, diabesity prevalence continues to rise globally, particularly in countries transitioning from low to middle-income economies (Saeedi et al., 2019). Excessive consumption of sugary and high-fat ultra processed foods, driven by hedonic rather than homeostatic mechanisms, has been compounded by increasingly sedentary lifestyles. Addressing the brain’s role in integrating hedonic and homeostatic signals offers a promising approach to curbing the global diabesity epidemic (Berthoud et al., 2017; Campos et al., 2022). Despite the distinction between hedonic and homeostatic feeding—where hedonic feeding is driven by pleasure and homeostatic feeding fulfilling basic metabolic needs—their overlapping neural circuits make them difficult to separate functionally and anatomically (Rossi and Stuber, 2018; Ahn et al., 2022).

The nucleus tractus solitarius (NTS), a complex structure in the dorsomedial medulla, plays a central role in integrative processes, including feeding. The NTS exhibits remarkable neurochemical diversity, with most neuroactive substances identified in the central nervous system also present in the NTS, where they function as classical neurotransmitters and/or neuromodulators. The NTS receives peripheral inputs from cranial nerves, notably vagal afferent projections, as well as signals from the glossopharyngeal, facial, and trigeminal nerves. Gustatory and somatic afferents from the oropharyngeal region map to the rostral NTS, while visceral afferents from the cardiovascular, digestive, respiratory, and renal systems terminate in the caudal NTS (Jean, 1991).

The NTS in turn maintains extensive connections with multiple central structures, directly projecting to regions involved in affective, appetitive, cardiorespiratory, and neuroendocrine functions (Beckstead et al., 1980; Geerling et al., 2006; Herbert et al., 1990; Holt, 2022; Loewy and Burton, 1978; Quillet et al., 2023; Rinaman, 2010; Horst et al., 1989). These connections include short pathways to bulbo-ponto-mesencephalic structures (e.g., the parabrachial nucleus, cranial nerve motor nuclei, ventrolateral reticular formation, and raphe nuclei) and longer pathways to the spinal cord, hypothalamus, and limbic structures (Jean, 1991).

Additionally, the NTS receives central top-down inputs, including direct projections from the cerebral cortex (Gasparini et al., 2020; Holt et al., 2019; Van Der Kooy et al., 1984; Whitehead et al., 2000). Most structures receiving projections from the NTS reciprocally project back, forming a closed-loop regulatory system. This design highlights the NTS’s pivotal role in autonomic and neuroendocrine functions, integrating somatic and autonomic responses to influence and regulate moment to moment behavior to and from all major organ systems.

Finally, depending on their topography within the NTS, distinct subpopulations of neurons can either potently reduce food intake (caudal ventral NTS) with or without the adverse hedonic affect, or increase hunger (rostral NTS neurons) (Gasparini et al., 2024). Interestingly, multiple NTS neuronal populations can cumulatively suppress food intake (Qiu et al., 2023), and oral, as well as visceral feedback to NTS can induce sequential appetite suppression (Ly et al., 2023).

The NTS overlaps sympathetic and vagal afferents (Tjen-A-Looi et al., 1997), both involved in type 2 diabetes pathophysiology (Zhang et al., 2024; Zsombok et al., 2024; Chen et al., 2024), as well as spinal cord connections through the cervical plexus (Stornetta et al., 2016). Therefore, optimal neuromodulation of the NTS through combined stimulation of the vagus nerve (caudal NTS) and the trigeminocervical complex (rostral NTS), as well as upper cervical spinal roots warrants assessment in optimizing clinical outcomes of type 2 diabetic patients and their associated co-morbidities—notably if mediated via minimally invasive modalities (Guglielmi et al., 2023). Interestingly, the outer ear is the only superficial organ anatomically innervated by the vagus cranial nerve (auricular peripheral branch nerve or ABVN), the trigeminal cranial nerve (via the auriculotemporal peripheral nerve or ATN), and the distal branches of the upper C2 and C3 cervical spinal roots (namely lesser occipital and greater auricular peripheral nerves respectively, LON and GAN) (Mercante et al., 2018). Moreover, aVNS stimulation alone is already a safe emerging minimally invasive neuromodulation therapy being explored in a wide range of diseases (Gerges et al., 2024; Farmer et al., 2021).

Given the interplay between autonomic dysfunction, metabolic imbalance, and chronic inflammation in T2DM, a neuromodulatory approach targeting both vagal and trigeminocervical pathways holds significant therapeutic promise. By simultaneously engaging these systems, this dual-modality stimulation may provide superior clinical benefits compared to single-pathway interventions.

Indeed, a longitudinal single center RCT by Madhuchander et Gurunath (NCT03540446; abstracts from the 22nd Annual Meeting of the North American Neuromodulation Society 2019) validated that auricular Percutaneous Electrical Neuro-Stimulation (PENS) stimulating the ATN, ABVN and LON peripheral cranial nerves could successfully improve DPN in all active-PENS patients who completed the 12 week-study (alternating stimulation on/off every 2 h—on a 1 week on/off basis). As the patients’ resilience in participating may explain this success (26 out of 89 patients dropped-out), we launched an observational study to question the efficiency of a similar combined peripheral stimulation, implanted during a shorter duration (less than 1 month), among the most severe, underserved DPN populations: Native Americans. Given the growing burden of T2DM and its complications, exploring innovative, non-pharmaceutical interventions is crucial. This pilot observational study pioneers the assessment of short-lived minimally invasive auricular neuromodulation in patients with severe DPN and T2DM.

## Materials and methods

### Design

Our study design was based upon the results of an unpublished Indian double-blind, randomized, placebo-controlled longitudinal trial (NCT03540446) assessing the significant effect of combined percutaneous electrical auricular neuro-stimulation of the trigeminal, vagal and superficial cervical complex in DPN. The latter study was presented at the 22nd annual meeting (Jan 17–20, 2019) of the North American Neuromodulation Society but was never published because of the death of one of the authors during the COVID pandemic. Remarkably, this yet-to-publish study showed a significant decrease of the DPN and even a quadratic reduction of pain in patients who also experienced good glycemic control (16 out of 43) in comparison with 20 placebo-treated patients (*p* < 0.001), supported by an analgesic requirement decrease of 80% versus only 7% in the placebo group. As the major drawback of this study was its length (12 weeks), a putative dropout factor since the percutaneous device used (First ReliefTM, DyAnsys Inc., San Mateo, CA, USA) requires a weekly visit of the ambulant patients (the stimulation is on for 1 week followed by 1 week off), we wanted to assess the effect of a minimally invasive implanted device (NS100, Neurosolutions100 Inc., Dallas, TX, USA) stimulating the same nerves during a time span of less than 1 month, minimizing the confounding effect of the resiliency of the patients. As we divided the time span of the stimulation by 2 (3 weeks instead of 6 weeks of stimulation within the 12-week follow-up), we wanted to double the size of the treated population, hence we needed a population of at least 86 patients, because of patients lost to follow-up. As the size of this population was relatively high and as the Indian double-blind, randomized, placebo-controlled had already shown significant efficiency of the combined peripheral stimulation, we decided to start with an observational study to assess if a shorter duration of stimulation could also improve DPN, before planning a double-blind randomized controlled study.

Thus, 91 adult Native Americans were included in a retrospective, non-controlled, observational study, to receive a combined aVNS and TCC peripheral nerve stimulation in the routine course of clinical care for severe DPN (and T2DM) from November 2022 to December 2023. Participants were selected from a single reservation to minimize the influence of genetic predisposition. Deidentified data analysis was approved and reviewed by the internal board of the Council of the Native American community. All patients provided informed consent, and authorization for the use of their data as part of a quality assurance program. No financial or other incentive was offered, nor paid to any patient for their participation.

Participants (meeting the inclusion/exclusion criteria) were prescreened for a primary Numeric Rating Scale (NRS) pain score 7 or greater (severe) on a scale of 1–10. Baseline pain assessments and mean capillary blood glucose measurements were obtained on the day of implant (day 0). All patients underwent implantation of a combined minimally invasive aVNS and TCC peripheral nerve stimulation device (NS100, Neurosolutions 100 Inc., Dallas, TX, USA) for 19 days and then explanted. NRS and mean capillary blood glucose levels were subsequently measured in a single outpatient clinic at 30-, 60-, and 90-days post-explant. Additional NRS pain scores and mean capillary blood glucose levels were then retrospectively analyzed up to 377 days.

### Eligibility

Inclusion criteria

- between Age 20–89 years old.
- diagnosis of T2DM and DPN with clinical documentation within last 3–6 months of mean capillary blood glucose > 126 mg/dL and severe diabetic neuropathic pain symptoms (e.g., “asleep numbness,” “prickling” or “stabbing,” “burning” or “aching” pain) with NRS score of 7 or greater
- history of failure of at least 2 drug classes (at adequate doses) and 1 or more non pharmacologic modality (e.g., physical therapy, TENS…) within the preceding 3–6 months.
- willing to undergo combined minimally invasive neurostimulation implantation
- willing to follow-up on site to record NRS pain scores and mean capillary blood glucose for 90 days
- provide informed consent

Exclusion criteria

- any bleeding diathesis
- pregnancy
- implantable cardiac devices (i.e., pacemaker, loop recorder, defibrillator)
- cardiac monitoring devices (i.e., halter monitor)
- indwelling/implantable stimulator devices (i.e., deep brain stimulator, permanent spinal cord, bladder stimulator)
- inability to give informed consent
- subject with automated insulin dosing (AID) systems.

### Screening, implantation and follow-up

**Figure 1:**
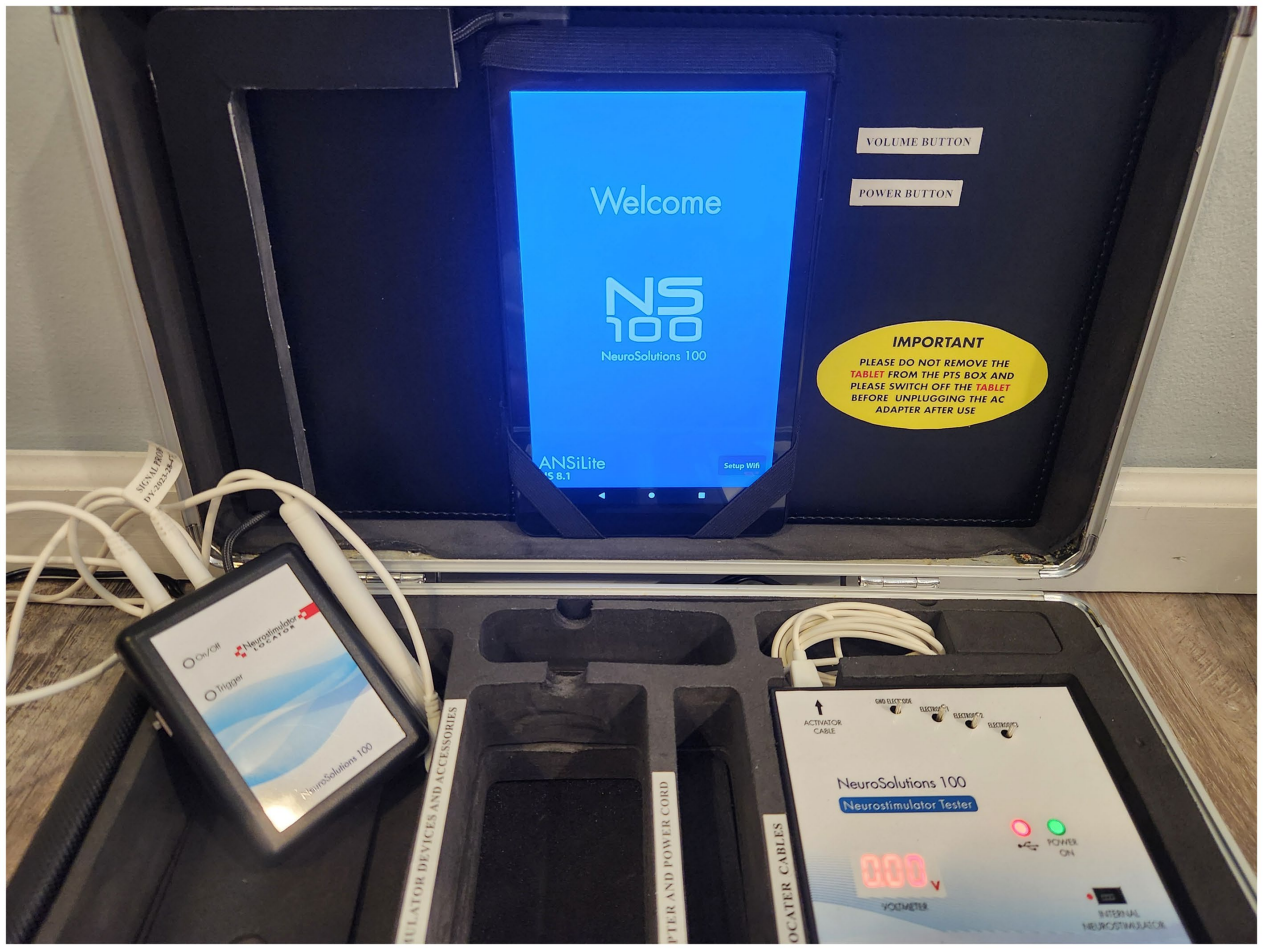
Patient Programming Technical Unit (PTU, Neurosolutions100 Inc., Dallas, TX, USA). Real time, closed loop, neural impedance drop feedback and alternating fixed and sweep wave current/power density patterns are proprietary precision neural localization and electrical field programming software platforms required to objectively identify, implant and program the NS100 Vagal and Trigeminocervical complex peripheral nerve stimulating device.

At screening and prior to implantation, the patient was provided informed consent including the exact nature of the analysis, known side effects, risks involved, other applicable treatment options, the visits and procedures required, and the option to withdraw without reason or prejudice to future care. The NRS pain score, mean capillary glucose and HbA1c were recorded (or estimated with Appendix 1) and complicity with the inclusion/exclusion criteria confirmed. If no contraindications were identified, 4 titanium electrodes were implanted on the auricular vagal (ABVN) and trigeminal cranial nerves (ATN), and the distal peripheral branches of the upper C2 and C3 cervical spinal roots (LON, GAN) utilizing real time, closed loop, neural impedance drop feedback. Each of the titanium electrodes were implanted 2 mm under the skin precisely on each of the respective nerves—after precise localization and programming using the proprietary software platforms in the patient programming technical unit. The day of implant was recorded as day 0. Low voltage stimulation impulses were then applied for 19 days with an alternating 2 h on/2 h off “fixed” and “sweep” current/power density pattern (1–100 Hz) before being explanted (Patient Programming Technical Unit (PTU), Neurosolutions100 Inc., Dallas, TX, USA, Figure 1). Follow up visits at 30-, 60-, and 90-days post explant further recorded NRS pain score, mean capillary blood glucose levels and HbA1c (or estimated with Appendix 1). Subjects were queried throughout for any adverse events. Finally, a random subset of patients was queried by a disinterested third party (a quality assurance, lay person liaison appointed by the internal review board of the Council of the Native American community) at the end of the virtual follow up period as to whether they were still requiring (yes or no) pain and/or diabetes medications.

### Endpoints

Primary outcome measures included:

1. NRS pain scores percentage improvement from implant (day 0) to 30-, 60- and 90-days post explant.

Secondary outcome measures included:

- Mean capillary blood glucose percentage improvement from implant (day 0) to 30-, 60- and 90-days post explant.
- HbA1c percentage improvement from implant (day 0) to 30-, 60- and 90-days post explant.
- Any Adverse Events (AE’s), occurring at any time during the implant, explant or follow-ups. (e.g., bleeding, infection, or any sensitivity to the implant).
- Continued pain and diabetes medication usage (yes or no) analysis (retrospectively) in a random subgroup (45 of 83).

### Statistical analysis

Analysis of sample characteristics for the groups was conducted to assess comparability of the samples. Categorical variables such as sex and biobehavioral data were assessed using Fisher’s exact test, but continuous variables such as age were assessed using two-tailed t tests. All reported *p* values are two tailed and considered significant at the 0.05 level, with standard assumptions (i.e., independence, heteroskedasticity, etc.) about continuous data applied. A post-hoc analysis with one-sided t-test, secondarily performed to estimate if the study was sufficiently powered based on observed effect sizes, still confirmed highly significant results (power above 80%) and validated the size of our population sample.

## Results

Ninety-one Native American patients with severe diabetic peripheral neuropathy pain (meeting the inclusion/exclusion criteria) underwent implantation, without any significant AE reported through the 90-day follow-up. Five patients experienced mild postural intensity changes at the electrode implantation site that resolved completely within 24–48 h. One patient experienced topical sensitivity to the surgical dressing which resolved within 24 h of implantation when the dressing was changed.

Eight patients were lost to follow-up, so statistical analysis was conducted on the remaining 83 patients (male 34, Female 49). All 8 lost-to-follow-up were equivalent responders systematically through explant (day 19). However, during the follow-up period one patient moved off reservation, and the remote geography and limited resource infrastructure contributed to the remaining seven patients being lost to a combination of: an inability to communicate (loss of cell phone/coverage without landlines); an unknown change in contact information; a loss of all transportation; and an inability to overcome financial and/or untenable, unspecified family constraints.

Average improvement at 30-, 60-, and 90-days after application of the aVNS and TCC peripheral nerve stimulator were followed for up to 8 months, or an average of 227 days. Medication usage over time was assessed by a retrospective follow-up call to all 83 patients asking the same single yes or no question (“Are you off all of your pain and diabetes medications?”). Unfortunately, only 45 of the 83 actually (randomly) answered the phone and 36 of those answered affirmatively. Hence, among the random subset of 45 patients, 80% reported discontinuing their medications after receiving combined aVNS and TCC neurostimulation treatment. Both the pain (DPN) and blood sugar improvements decreased most significantly within the first 90 days, and then remained at those lower levels through the end of the follow up period—reported as an average of 7.85 months of all 83 patients.

**Figure 2:**
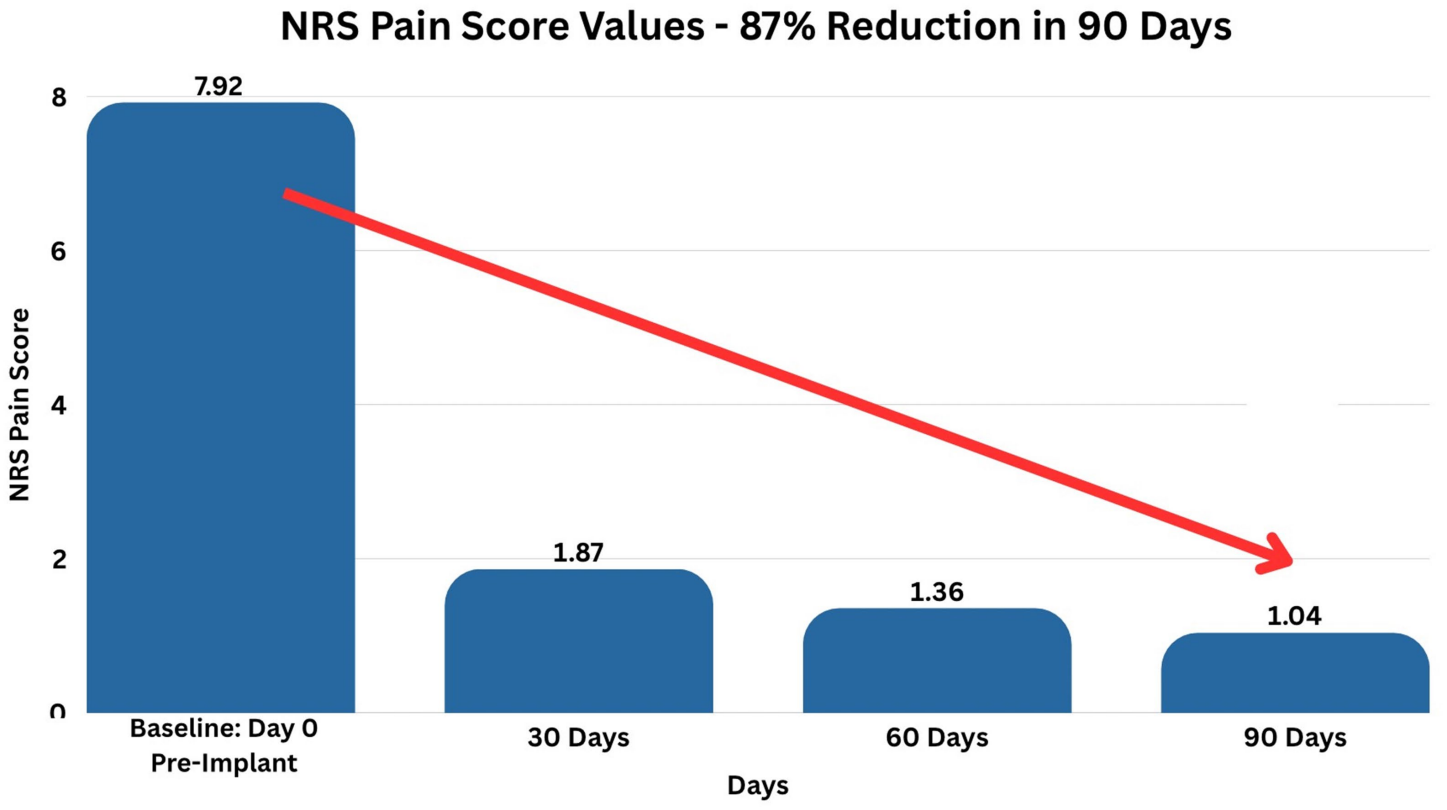
NRS pain score values. This figure denotes the reported Numeric Rating Scale (NRS) pain score values over 90 days. The average reported pain was reduced from 7.92 on day 0 to 1.04 on day 90, an overall 87% reduction.

DPN pain levels decreased, respectively, by 78, 83 and 87% at 30-, 60-, and 90-day follow ups (as shown in Table 1). As a consequence, the level and percent improvements in pain scores at 30, 60, and 90 days are remarkably statistically significantly different from baseline at the 5, 1, and 0.1% levels (Table 2). This dramatic 87% reduction of pain (from 7.92 on day 0 to 1.04 on day 90) is visually represented in Figure 2.

**Table 1:** Pain score values.

Baseline pain score	Time period	Pain score	Level difference	% Difference
7.92	30-day	1.87	−6.16	78%
7.92	60-day	1.36	−6.64	83%
7.92	90-day	1.04	−6.94	87%

This table reports the baseline pain levels, and the levels of pain reported and 30-, 60-, and 90-day follow ups.

**Table 2:** Pain improvements & one-sample, two-sided student’s t-test results.

Indicator	Mean	Lower bound	Upper bound	Standard error	t-test statistic	*p* Value
Pain level improvement, 30 days	−6.16	−6.56	−5.76	0.20	−30.74	8.949e−47***
Pain percent (%) improvement, 30 days	78%	74%	81%	0.02	40.57	4.838–56***
Pain level improvement, 60 days	−6.64	−7.06	−6.23	0.21	−32.07	3.619e−48***
Pain percent (%) improvement, 60 days	83%	79%	86%	0.02	43.85	1.081e−58***
Pain level improvement, 90 days	−6.94	−7.32	−6.56	0.19	−36.08	4.324e−52***
Pain percent (%) improvement, 90 days	87%	85%	90%	0.01	61.09	3.763e−70***

This table describes the level and percent improvements in pain scores at 30, 60, and 90 days. Level improvement reflects the subtracted difference between the baseline pain score and the follow-up pain score, as shown also in the fourth column of Table 1. Mean scores for the difference in levels and the percentage improvements were calculated, and standard one sample, two-sided student’s t-tests were run on each. Results were statistically significantly different from baseline at the 5, 1, and 0.1% levels. *** means very highly significant, *p* < 0.0001.

**Figure 3:**
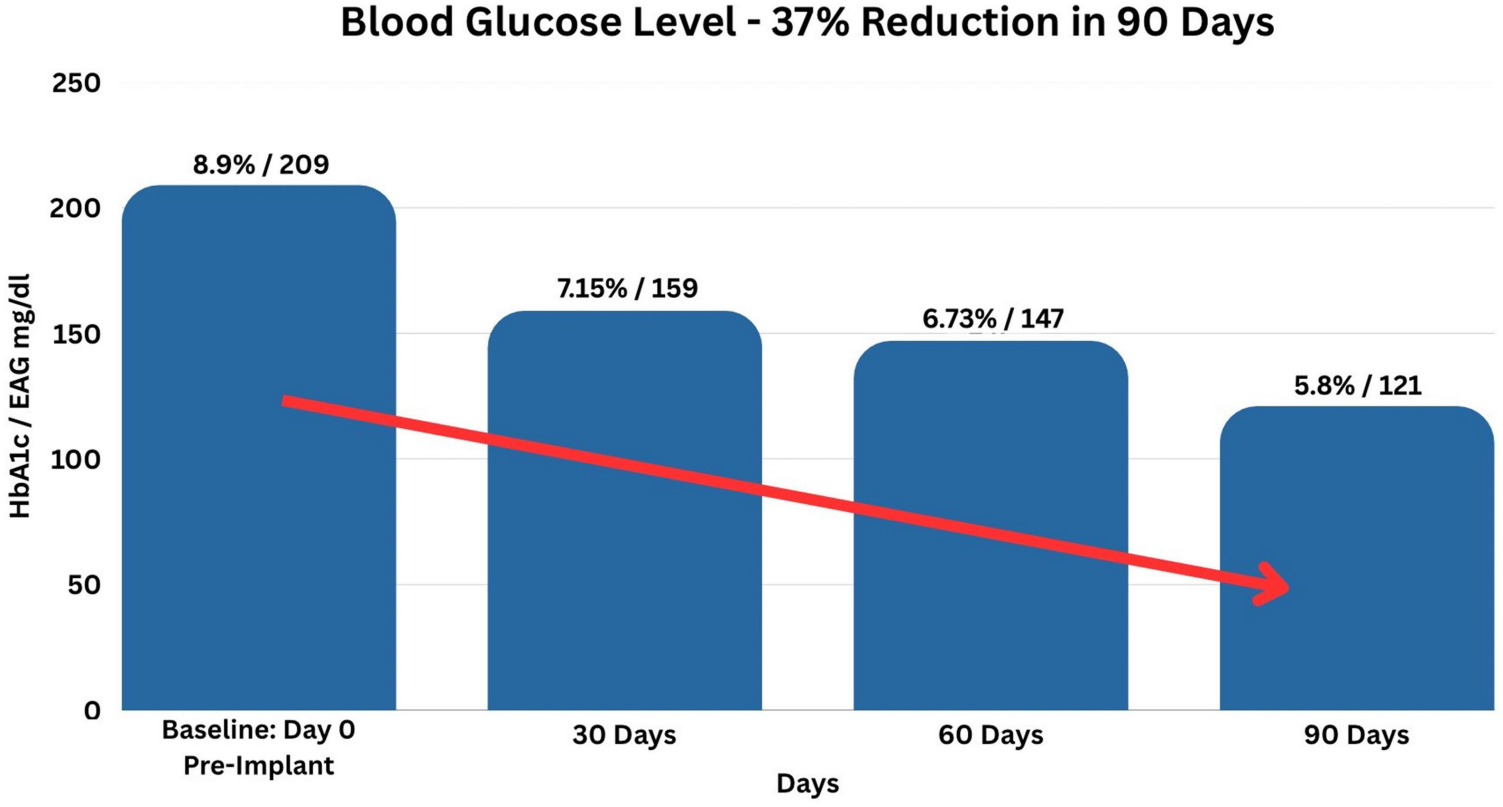
Blood glucose levels. This figure denotes average blood glucose levels over 90 days, which dropped from 8.9% or 209 mg/dL on day 0 to 7.15% or 159 mg/dL on day 30, to 6.73% or 147 mg/dL on day 60, to 5.8% or 121 mg/dL, a 37% overall reduction.

Regarding glycemia, glucose levels and percentage improvements for 30-, 60-, and 90-day follow-ups, respectively 19, 24, and 37%, are shown in Table 3. Then, Table 4 describes one-sample, two-sided student’s t-test results for the level and percent improvement in blood glucose measurements. In terms of both level and percentage improvements, results are statistically significant at the 5, 1, and 0.1% levels. Furthermore, by 90 days, the glucose mean was 121 mg/dL which is within normal glucose levels. As such, by 90 days, many patients no longer experienced hyperglycemia. Finally, Figure 3 displays the glycemic drop from 8.9% or 209 mg/dL on day 0 to 5.8% or 121 mg/dL at 90 days, a 37% overall reduction.

**Table 3:** Blood glucose levels.

Baseline blood glucose levels (HbA1c 8.9%)	Time of follow-up	Blood glucose level	Level difference	% Difference	HbA1c values
209	30-day	159	−50	19%	7.15
209	60-day	147	−63	24%	6.73
209	90-day	121	−89	37%	5.8

This table reports the baseline blood glucose levels mg/dL and levels and percentage improvements as well as HbA1c values (%) for 30, 60, and 90 day follow ups.

**Table 4:** Blood glucose improvements & one-sample, two-sided student’s t-test results.

Indicator	Mean	Lower bound	Upper bound	Standard error	t-test statistic	*p* Value
Blood glucose level improvement, 30 days	−50.32	−62.75	−37.88	6.25	−8.05	6.858e−12
Blood glucose percent (%) improvement, 30 days	19%	13%	25%	3%	6.52	6.156e−09
Blood glucose level improvement, 60 days	−62.64	−76.63	−48.64	7.03	−8.91	1.606e−13
Blood glucose percent (%) improvement, 60 days	24%	18%	30%	3%	8.17	4.502e−12
Blood glucose level improvement, 90 days	−88.97	−103.26	−74.69	7.17	−12.41	4.793e−20
Blood glucose percent (%) improvement, 90 days	37%	33%	41%	0.02	17.99	4.092e−29

This table describes one-sample, two-sided student’s t-test results for the level and percent improvement in blood glucose measurements following auricular nerve stimulation. In terms of both level and percentage improvements, results are statistically significant at the 5, 1, and 0.1% levels.

## Discussion

DPN is characterized by burning, tingling, or shooting pain that significantly impacts quality of life and increases healthcare utilization (Higgins et al., 2022). The medical management of DPN has primarily focused on symptom control and improved patient functionality. However, the complexity of this condition and variability in patient response necessitate a multimodal approach tailored to individual needs.

The first-line pharmacologic agents for managing DPN include antidepressants, such as serotonin-norepinephrine reuptake inhibitors (SNRIs) and tricyclic antidepressants (TCAs), as well as anticonvulsants like gabapentinoids (Rafiullah and Siddiqui, 2022; Attal, 2019). Duloxetine, an SNRI, has been widely studied and is approved by the U.S. Food and Drug Administration (FDA) for DPN (Rastogi and Jude, 2021). It works by modulating descending pain pathways in the central nervous system (CNS) (Boomershine et al., 2011). Similarly, pregabalin, a gabapentinoid, is FDA-approved for DPN and reduces neuropathic pain by binding to voltage-gated calcium channels, thereby attenuating neuronal excitability (Alles et al., 2020; Paul et al., 2022). Despite their efficacy, these agents often have dose-limiting side effects, such as dizziness, sedation, and gastrointestinal upset, which can reduce patient adherence (McCarberg et al., 2012).

In cases where first-line therapies fail to provide adequate relief, second-line agents, including opioids and topical treatments, are considered. Tapentadol, a dual-action opioid and norepinephrine reuptake inhibitor, has demonstrated efficacy in clinical trials but is associated with concerns about dependence and tolerance (Alshehri, 2023). Topical agents like 8% capsaicin patches and 5% lidocaine patches offer localized pain relief with minimal systemic side effects, making them particularly useful in patients unable to tolerate systemic medications (Elbeddini et al., 2024).

A new pharmacological class mimicking the actions of natural incretin hormones (principally glucagon-like peptide-1 (GLP-1) and glucose-dependent insulinotropic polypeptide (GIP)), potentiating both meal-stimulated insulin and glucagon secretion (Drucker and Holst, 2023), arouses keen interest for the treatment of T2DM and obesity. Besides GLP-1 receptor agonists (A-GLP1), on the market since 2005, newer multi-agonist drugs that use one molecule to activate multiple receptors are drawing attention, like tirzepatide (that stimulates both the GLP-1 and the GIP receptor), currently approved for T2DM. Studies on these multi agonist drugs suggest that activation of multiple receptors is more effective than activating A-GLP1 alone (Karagiannis et al., 2024). They could even prevent the onset of T2DM in people with excess weight or obesity (Jastreboff et al., 2025). The results of the SURMOUNT-1 trial, recently published in The New England Journal of Medicine found that after more than 3 years, nearly 99% of all participants who took tirzepatide were able to prevent type 2 diabetes, and 94% of participants with prediabetes taking tirzepatide were able to reverse their prediabetes and return their glucose levels to normal. In addition, research demonstrated that participants taking tirzepatide saw up to 23% body weight reduction, increased levels of HDL cholesterol, and reductions in waist circumference, blood pressure, and triglycerides throughout the study.

These results are promising, with the caveat that tirzepatide and other drugs in the same class offer risks along with benefits and may not be suitable for everyone. Indeed, for instance, the increasing use of A-GLP1 has recently been linked to several side effects including not only gastrointestinal disorders, hypotension, syncope, arthritis disorders, nephrolithiasis, interstitial nephritis drug-induced pancreatitis (Xie et al., 2025), but also severe ophthalmological complications (Katz et al., 2025) compared to usual care. As retrospective studies cannot establish a causal link between exposure to A-GLP1 and the complications observed, if this link exists, it is likely to result rather from a rapid correction of hyperglycemia than from a direct toxic effect of these medications. By comparison, combined aVNS and TCC stimulation is likely to be a safer option since it takes 90 days to normalize glucose blood levels.

Spinal cord stimulation offers promising results in refractory cases (Wang et al., 2022; Henson et al., 2021) but is relatively costly and carries the risk of operating on the spinal column. Therapies such as cognitive-behavioral therapy (CBT) and physical therapy have demonstrated safety and varying degrees of effectiveness in improving pain and functionality (Higgins et al., 2022; Shi and Wu, 2023; Li et al., 2023; Akyuz and Kenis, 2014; Jahantigh Akbari et al., 2020), albeit with less pronounced benefits in DPN than in our pilot.

Indeed, notably through GLP-1 signaling, VNS by itself can improve T2DM, weight gain and DPN (Longo et al., 2023; Dai et al., 2020; Dhanapalaratnam et al., 2024). It is well-recognized that VNS, with appropriate parameters, can improve diabetes mellitus in animal models (Payne et al., 2022; Waataja et al., 2023). The vagus nerve, a key regulator of glucose homeostasis, promoting insulin secretion and improving tissue insulin sensitivity by counteracting sympathetic overactivity and reducing systemic inflammation (Sorski and Gidron, 2023)—a major driver of insulin resistance and DPN, is both anatomically (Xiong et al., 2023) and functionally (Yu et al., 2020) down-impacted in T2DM patients with DPN. Additionally, VNS is known to reduce food intake and body weight by delaying gastric emptying. Interestingly, diet-induced shift in the gut microbiome is likely to directly induce DPN, according to a recent bi-directional two sample Mendelian randomization study (Xie et al., 2024). Given that electrical stimulation of the AVBN is able to both change gut microbiota composition and improve “abdominal pain, functioning and catastrophizing” in adolescents (Castillo et al., 2023) as well as to activate enteric neurogenesis and synaptic efficiency in a rodent model (Soylu et al., 2025), it is likely that a limited duration of stimulation of this essential component of the microbiota-gut-brain axis is sufficient to long lastingly reshape and optimize neural networks involved in metabolism and body weight (Obst et al., 2022; Vaughn et al., 2017), notably via the endocannabinoid system (Wood et al., 2024; DiPatrizio, 2021; Patterson et al., 2016). Finally, it is not surprising that VNS could also improve DPN in our trial since it can stimulate VEGF-A synthesis (Lv et al., 2018) and even promote oligodendrogenesis, facilitating the restoration of myelin sheaths in animal models (Huang et al., 2024).

Transcutaneous auricular VNS (taVNS) seems efficient by itself, since a single session of 1 h-stimulation was able to improve both neuropathic pain (subjectively) and HRV (objectively) (Wong et al., 2025). In this controlled pilot trial, the sham stimulation was applied on the ear lobe, the latter not being supplied by the auricular branch of the vagus nerve but by the GAN and LON, originating from the C2 and C3 spinal nerves (Peuker and Filler, 2002; Mercante et al., 2018). Surprisingly and very interestingly, all symptoms decreased post active and sham taVNS, with greater mean change for the active condition, but the difference was not statistically significant between conditions (*p* values >0.11 across all symptoms). HRV increased during sham taVNS, a stimulation of the trigeminocervical complex, indicating that this was not a true sham stimulation, but an active stimulation of a portion of the TCC complex.

Remarkably, our findings surpass outcomes reported by a recent longer randomized controlled study assessing the efficiency of transcutaneous cervical VNS alone on continuous glucose monitoring in 145 diabetic patients with DPN (including 75 T2DM) for either 1 week (4 daily stimulation of 2 min) or 8 weeks (2 daily stimulations of 2 min) (Kufaishi et al., 2025). These discrepancies of efficiency between the two noninvasive vagus nerve stimulation techniques (i.e., cervical and auricular) could possibly result only from the differences between the protocols used, corroborated by the significant difference in number of vagal fibers between the ear and the neck.

Nevertheless, targeting both the vagus and trigeminal nerve pathways, as in our trial, is likely to yield a more robust therapeutic effect. This synergy may be attributed to the broader engagement of central autonomic and inflammatory control centers. The concurrent stimulation of the TCC may further amplify these effects, as this pathway interacts with the NTS, a central hub for autonomic and metabolic regulation. Notably, cross-talk between vagus and trigeminal nerve stimulation have been reported (Möller et al., 2020; Peng and May, 2022). Both VNS (Brougher et al., 2021; Diepenbroek et al., 2024; DiPatrizio, 2021) and TCC stimulation (Xu et al., 2023; Leimuranta et al., 2018; Zorrilla et al., 2024) can modulate human motivation and behaviors by impacting the dopaminergic and endocannabinoid systems. Moreover, stimulating the TCC could counteract the impact of the estrous cycle on food intake in females without modifying their hormonal status by potentiating the estrogen-dependent signaling (Benchoula et al., 2024; Klen and Wolkoff, 2004) involved in both antinociception (Spekker et al., 2022; Nag and Mokha, 2016) and satiety signal (Huang and Raybould, 2020 for a review).

Thus, combining trigeminocervical and vagal neuromodulation stimulates multi-level interplaying closed loops, potentiating their single action, which might explain the impressive changes observed in our observational study, long after stopping the stimulation. There are two closed loop feedback loops noted. The first is the natural closed loop physiology that is being activated by the combined TCC and VNS within the brainstem and more precisely in the NTS. That is, by stimulating these three entries (auricular vagus nerve, trigeminal and cervical neural complexes) instead of one (the vagus nerve alone), we take advantage of the innate closed loop to regulate more efficiently and for a long-term period blood sugar level and DPN (discussed in the discussion). The second is the proprietary closed loop impedance drop software platform referenced above within the PTU (Figure 1) that objectively localizes each of the individual nerves of interest to be implanted. However, as our combined stimulation of aVNS and TCC may only partly induce the large size improvements reported due to several confounding factors, (namely the placebo effect, the regression to the mean, concomitant care changes including medication during the study, the natural history of the cohort), further double-blind, controlled, randomized trials are definitely needed to assess the potential causal inference.

This dual-pathway approach also addresses the underlying vascular and bioenergetic dysfunction associated with DPN. By enhancing mitochondrial efficiency and restoring vascular endothelial growth factor (VEGF-A) expression, neuromodulation improves peripheral nerve function and reduces pain. The rapid reduction in neuropathic pain observed in our analysis, along with sustained glucose control, may reflect the synergy between metabolic and neurological improvements.

Provided this condition is met, neurostimulation could become transformative in T2DM associated with obesity management, for cost-effectiveness purposes. Diabetes is a major healthcare crisis affecting more than 422 million people worldwide with 38 million in the USA—15% of which have painful diabetic neuropathy. Painful DPN management alone increases both the direct cost of treatment up to threefold, and leads to work absence, change in employment and disability (Tesfaye et al., 2010). Furthermore, only 32.15% (8.2 million) of 25.5 million adults with diagnosed diabetes are employed. This equates to $28.3 billion in lost employment, $35.8 billion from presenteeism, $32.4 billion due to premature mortality, $5.4 billion in work absences, and $4.4 billion from lost productivity. At these current trends, the overall cumulative cost of diabetes, pre-diabetes and its associated co-morbidities will consume all national healthcare expenditures within the next 5–10 years (Dyess, 2023; Lin et al., 2018; Cawley et al., 2021). Remarkably, the minimally invasive nature of auricular neurostimulation offers a safer, more accessible and cost-effective therapeutic alternative, potentially increasing patient adherence and treatment scalability. The significant improvements in pain and glucose control—which lasted an average of 227 days after only 19 days of treatment (in comparison to lifelong drug treatment)—along with the associated physical and mental relief, may support reintegration into the workforce, thereby reducing both personal, mental health, and societal economic burdens related to DPN and T2DM. Examining the cost-effectiveness of this therapy, particularly in populations disproportionately affected by T2DM, such as Native American communities, could support its broader adoption and has to be assessed in future double-blind, randomized, placebo-controlled trials, using lab-based HbA1c, validated diabetes control questionnaires, and rigorously tracking medication changes ideally with prescribing data and structured interview.

## Conclusion

The significant improvements observed in our observational study include an 87% reduction in pain, a 37% decrease in mean blood glucose, a reduction in HbA1c from 8.9 to 5.8% and a random subset that discontinued 80% of all pain and diabetes medications; all sustained for an average of 7.85 months; surpassing prior outcomes achieved with cervical VNS alone.

Thus, this open label pilot positions combined minimally invasive aVNS and TCC stimulation as a transformative, non-pharmacologic alternative capable of addressing both the DPN and diabesity crisis, as well as the intertwined pathophysiology of diabetes, neuropathy, and metabolic dysfunction. As such, this approach has the potential to restore autonomic balance and redefine the standard of care for diabetic patients by reducing the long-term societal, mental health, and economic burdens of the disease. Further double-blind randomized controlled trials are needed to confirm, better elucidate the mechanisms and biomarkers (i.e., Heart Rate Variability, Laser Evoked Potentials), and establish standardized protocols for broader clinical application.

## Data Availability

The raw data supporting the conclusions of this article will be made available by the authors without undue reservation.
