# Supplementary figures and images for: Combined minimally invasive vagal cranial nerve and trigeminocervical complex peripheral nerve stimulation produces prolonged improvement of severe painful peripheral neuropathy and hyperglycemia in type 2 diabetes
Source: Front Neurosci. 2025 Aug 26;19:1644961. doi: 10.3389/fnins.2025.1644961 (PMC12418259; doi:10.3389/fnins.2025.1644961)

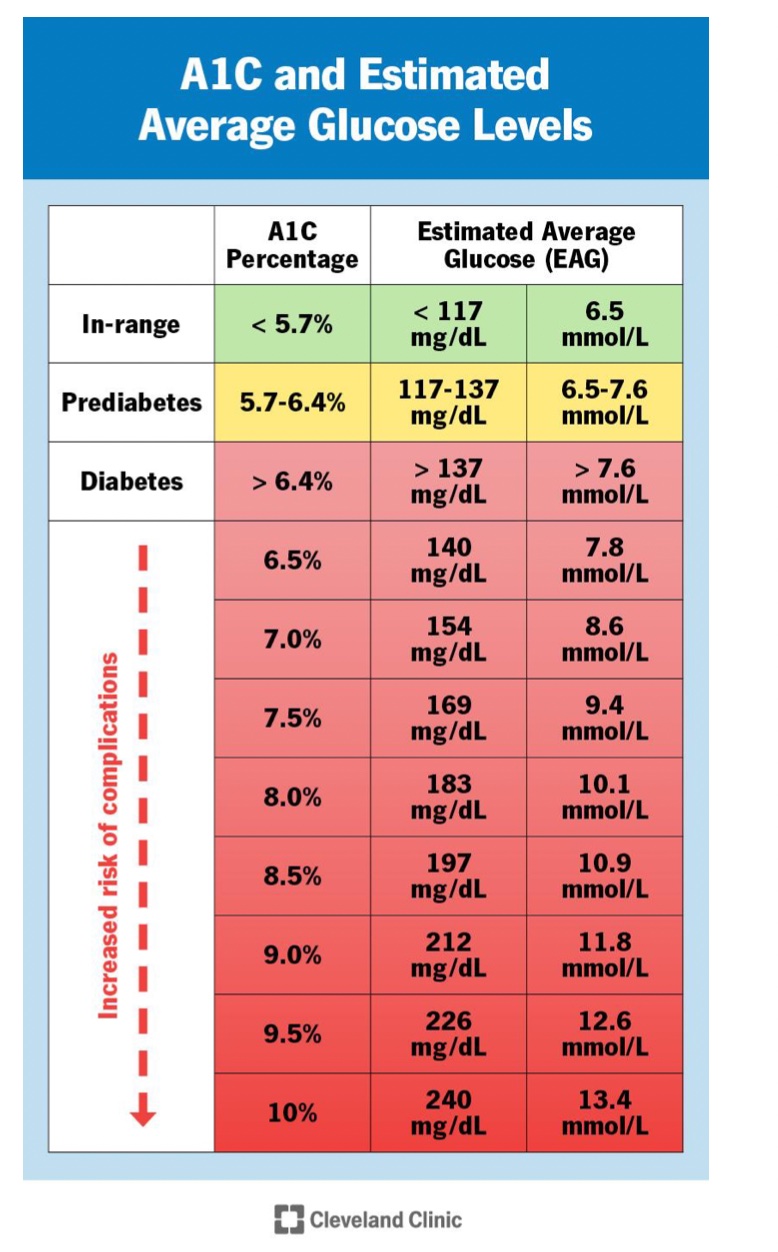

Supplement: Supplementary file 1 [file Image_1.jpeg]
